# Legal concerns in health-related artificial intelligence: a scoping review protocol

**DOI:** 10.1186/s13643-022-01939-y

**Published:** 2022-06-17

**Authors:** Michael Da Silva, Tanya Horsley, Devin Singh, Emily Da Silva, Valentina Ly, Bryan Thomas, Ryan C. Daniel, Karni A. Chagal-Feferkorn, Samantha Iantomasi, Kelli White, Arianne Kent, Colleen M. Flood

**Affiliations:** 1 University of Ottawa Faculty of Law, Fauteux Hall, 57 Louis-Pasteur Private, Ottawa, ON K1N 6N5 Canada; 2 The Royal College of Physicians and Surgeons of Canada, Toronto, Canada; 3 School of Epidemiology and Public Health, University of Ottawa, Ottawa, Canada; 4 The Hospital for Sick Children, Toronto, Canada; 5 University of Toronto, Toronto, Canada; 6 University of Ottawa, Ottawa, Canada

**Keywords:** Health law, Artificial intelligence, Machine learning, Health, Scoping review

## Abstract

**Background:**

Medical innovations offer tremendous hope. Yet, similar innovations in governance (law, policy, ethics) are likely necessary if society is to realize medical innovations’ fruits and avoid their pitfalls. As innovations in artificial intelligence (AI) advance at a rapid pace, scholars across multiple disciplines are articulating concerns in health-related AI that likely require legal responses to ensure the requisite balance. These scholarly perspectives may provide critical insights into the most pressing challenges that will help shape and advance future regulatory reforms. Yet, to the best of our knowledge, there is no comprehensive summary of the literature examining legal concerns in relation to health-related AI. We thus aim to summarize and map the literature examining legal concerns in health-related AI using a scoping review approach.

**Methods:**

The scoping review framework developed by (J Soc Res Methodol 8:19-32, 2005) and extended by (Implement Sci 5:69, 2010) and the Preferred Reporting Items for Systematic Reviews and Meta-Analysis extension for scoping reviews (PRISMA-ScR) guided our protocol development. In close consultation with trained librarians, we will develop a highly sensitive search for MEDLINE® (OVID) and adapt it for multiple databases designed to comprehensively capture texts in law, medicine, nursing, pharmacy, other healthcare professions (e.g., dentistry, nutrition), public health, computer science, and engineering. English- and French-language records will be included if they examine health-related AI, describe or prioritize a legal concern in health-related AI or propose a solution thereto, and were published in 2012 or later. Eligibility assessment will be conducted independently and in duplicate at all review stages. Coded data will be analyzed along themes and stratified across discipline-specific literatures.

**Discussion:**

This first-of-its-kind scoping review will summarize available literature examining, documenting, or prioritizing legal concerns in health-related AI to advance law and policy reform(s). The review may also reveal discipline-specific concerns, priorities, and proposed solutions to the concerns. It will thereby identify priority areas that should be the focus of future reforms and regulatory options available to stakeholders in reform processes.

**Trial registration:**

This protocol was submitted to the Open Science Foundation registration database. See https://osf.io/zav7w.

## Background

Innovation in medicine offers tremendous hope but appropriate regulations are needed to realize this hope in healthcare and public health settings while minimizing attendant risks. The advent of health-related artificial intelligence (AI) provides an example of this need for regulation that fosters the requisite balance of potential benefits and pitfalls. Health-related AI is the topic of significant debate across multiple fields. Many portend that AI will improve healthcare systems by, e.g., increasing the accuracy of diagnoses, improving the efficiency of healthcare delivery, or mitigating human biases (e.g., [1, 2]). At the same time, there are understandable concerns that AI will undermine the patient-provider relationship, contribute to the deskilling of providers, undermine transparency, misdiagnose or inappropriately treat because of errors within AI decision-making that are hard to detect, exacerbate existing racial or societal biases, or introduce algorithmic bias that will be hard to detect (e.g., [3]). Opinions are, of course, not starkly polarized. Many simultaneously recognize AI’s promise and maintain concerns about its widespread adoption. Yet, these considerations highlight the need for regulations or other forms of governance (law, policy, ethics) that help countries leverage AI’s potential benefits while minimizing any attendant risks.

The use of health-related AI will intersect with the law in several ways. First, there are questions about whether existing laws will address identified concerns with health-related AI, such as the following:

i. Whether medical device regulation, medical malpractice laws, product liability laws, and professional self-regulation and accreditation will adequately attend to the possibility of error on the part of AI;
ii. Whether existing rules concerning the attribution of liability for medical error are appropriate for when AI tool recommends—or even performs—a course of harmful treatment and how liability should be attributed as between healthcare professionals and AI developers and manufacturers;
iii. Whether existing anti-discrimination and human rights laws can attend to the problem of algorithmic bias in which AI tools inappropriately produce different outcomes for historically disadvantaged groups;
iv. Whether existing privacy laws sufficiently protect patients given AI’s big data needs and the fact that machine learning (ML) tools, for example, will collect data in real time;
v. Whether existing laws and policies relating to data governance are sufficient to enable AI innovators to have access to representative training data sets so as to appropriately include historically underrepresented populations;
vi. Whether existing laws of informed consent are sufficiently robust to protect patients when clinicians choose to use AI in diagnosis and treatment.

Satisfactory responses are pressing as AI use increases in healthcare and public health settings. AI tools can already read medical images and reports more quickly and accurately than human counterparts [1, 2]. The U.S. Food and Drug Administration has approved AI tools for diagnosing strokes and brain bleeds, detecting atrial fibrillation, and interpreting brain MRIs; hospitals could also use other existing AI tools to assess patients’ risk of readmission and respond accordingly [1, 2]. Health-related AI is also used in Canada, including for triage purposes and to respond to COVID-19 [4]. The development of ML as a subset of AI that uses large data sets to “make predictions and solve problems … without being explicitly programmed” [5] has produced increasingly accurate health-related applications that can “learn” from real-world data over time and improve healthcare systems. As these technological innovations continue, there is a clear need to examine whether existing laws are up to the task of ensuring beneficial health-related AI tools can be deployed in real-world settings while minimizing legitimate concerns about, e.g., bias or privacy, that may arise.

Depending on one’s conclusions regarding the sufficiency of existing regulations to address concerns associated with the use of health-related AI, the next question is what kind of regulatory reform is required. Overly onerous regulations could stifle innovation, minimizing AI’s potential to realize health-related benefits. At the same time, a lack of regulation or inadequate regulations may lead to widespread harm resulting in a loss of trust in health-related AI (from patients themselves and healthcare professionals) and, perhaps, much more arduous regulation.

Despite the importance of law for the successful implementation of AI in health settings, there is no systematic overview of the legal concerns raised by the use of health-related AI. A preliminary search of MEDLINE®, Cochrane Library, PROSPERO, and JBI Evidence Synthesis was conducted; no current or underway systematic reviews or scoping reviews on this topic were identified. Two published scoping reviews were identified that survey ethical concerns raised by the use of health-related AI [5, 6] with one of the two taking a narrower focus on ethical issues concerning the disabled [6]. Although ethical issues can overlap with legal issues in some cases, legal concerns and legal responses are important to understand in their own right. For example, the law has an important claim to *democratic legitimacy* and failing to include legal issues means a failure to include concepts, such as patient autonomy, as articulated in sources of public and private law (e.g., Ontario’s *Personal Health Information Protection Act*). To illustrate with this one example, some may think that a consideration of ethical issues will capture concerns relating to consent to treatment, but a legal requirement for informed consent (as in Ontario’s *Health Care Consent Act*) can differ in its nature and impact. Further, although lawyers disagree on what the law should be, there is less room for disagreement than with respect to many ethical issues. Precedent (higher courts binding lower courts) helps create consistency in interpretation notwithstanding personal views on what the law should be.

A range of reports from different jurisdictions has identified areas where law reform may be needed to respond to problems in health-related AI ([4, 7–13]). Scholars have produced general overviews of the use of AI in healthcare that make claims with legal implications. For instance, Eric Topol’s well-known studies discuss concerns about the risk of AI tools causing harm to patients that implicate questions about AI safety and efficacy regulation and about both contractual and tort liability for harm [1, 2]. Other scholars have produced ethical analyses of health-related AI with possible legal implications. For instance, Alessandro Blasimme and Effy Vayena [14] raise concerns regarding AI safety, bias, and informed consent that may require legal responses of some kind. Mark Henderson Arnold [15] raises similar concerns, as well as questions about what to do when providers become too reliant on AI, which raise further liability questions. A relatively small number of works have articulated views on the most pressing legal concerns and possible best practices for approaching them (e.g., [3, 16]). Yet, there is no systematic overview of the legal concerns. As discussed above and further below, law and ethics overlap in important ways but legal and ethical issues can and should be distinguished where possible. A scoping review of paradigmatic and self-identified legal issues that identifies which concerns experts from different disciplines view as most important will enable better analysis of the adequacy of laws in various jurisdictions and what reforms are required (if any).

### Objective

We aim to systematically map legal concerns that are identified in health-related AI and the extent to which they are prioritized across multiple relevant disciplines, namely law, medicine, nursing, pharmacy, other healthcare professions (dentistry, nutrition, etc.), public health, computer science, and engineering. In keeping with the central purposes of a scoping review approach, we aim to examine the extent, range, and nature of research activity across the disciplines; to summarize and disseminate research findings to relevant stakeholders; and to identify research gaps in the existing literature. The scoping review will be conducted in accordance with the framework developed by Arksey and O’Malley [17] and extended by Levac et al. [18] and aligned protocol reporting to the PRISMA-P checklist [19]. It will thus include 6 stages: (1) Identifying the Research Question(s); (2) Identifying Relevant Studies; (3) Study Selection; (4) Charting the Data; (5) Collating, Summarizing, and Reporting Results; and in this case, (6) Stakeholder Consultation.

### Stage one: identifying the research question(s)

Through iterative discussions, research team members met to discuss and refine the research question(s). To ensure diverse perspectives and representation, the team included expert clinicians, AI innovators, legal researchers, ethics experts, and a member with scoping review expertise. From these discussions, we decided that our aim to map the nature, extent, and range of literature examining legal concerns in health-related AI. To this end, our research question(s) are as follows:

#### Primary question

1. What is known from the literature regarding legal concerns in health-related AI?

#### Secondary questions

1. Are the legal concerns identified explicitly prioritized?
2. Do different disciplines identify, represent, or prioritize legal concerns differently?

### Stage two: identifying relevant studies

Guided by two expert information specialists, we will develop a highly sensitive search strategy to identify relevant records. A preliminary pilot search of MEDLINE® and HeinOnline was conducted to pilot test the draft search and its ability to identify key articles. Titles, abstracts, keywords, and Medical Subject Headings (MeSH) in MEDLINE® of the key articles were analyzed to develop the final search strategy. See Table 1 for details. The MEDLINE® search was peer-reviewed by an independent information specialist using the Peer Review of Electronic Search Strategies (PRESS) checklist [20]. This original search strategy will be adapted to search additional medical, legal, and multidisciplinary databases as applicable. Searching will be conducted in the following electronic databases: MEDLINE® (Ovid), EMBASE (Ovid), HeinOnline Law Journal Library, Index to Foreign Legal Periodicals (HeinOnline), Index to Legal Periodicals and Books (EBSCO), Web of Science, Scopus, and IEEE Xplore. Given the complexity of searching (e.g., its multidisciplinary), the strategies will be augmented by hand-searching reference lists of all relevant, full-text records to identify additional sources [21]. Search results will be exported into a proprietary review software program (Covidence) to facilitate review processes and manage each stage of the review (e.g., de-duplication, eligibility assessment).

**Table 1:** MEDLINE® search strategy

#	Search	Results
1	artificial intelligence/	26391
2	machine learning/	16485
3	deep learning/	6249
4	supervised machine learning/	931
5	unsupervised machine learning/	506
6	natural language processing/	4799
7	neural networks, computer/	31313
8	robotics/	22242
9	((machin* or artific* or comput* or robot* or automat*) adj3 intelligen*).ti,ab,kf.	16925
10	((assist* or augment* or autonomous) adj1 intelligen*).ti,ab,kf.	353
11	((machin* or deep or transfer or hierarchical) adj2 learning).ti,ab,kf.	65348
12	(autoML or robot* or droid* or android* or telerobot* or tele-robot* or (remote* adj2 operat*)).ti,ab,kf.	57138
13	natural language processing.ti,ab,kf.	4541
14	neural network*.ti,ab,kf.	62994
15	(comput* adj2 (reason* or vision* or knowledg* or cogniti*)).ti,ab,kf.	8819
16	(perceptron* or connectionis*).ti,ab,kf.	4215
17	legislation as topic/	15972
18	legislation, hospital/	2455
19	legislation, medical/	16581
20	medical device legislation/	271
21	international health regulations/	66
22	legislation, nursing/	3161
23	legislation, pharmacy/	1266
24	privacy/	6934
25	jurisprudence/	29909
26	confidentiality/	23828
27	contracts/	3304
28	informed consent/	37305
29	informed consent by minors/	231
30	third-party consent/	3776
31	parental consent/	3298
32	intellectual property/	1602
33	patents as topic/	10109
34	copyright/	682
35	international law/	100
36	legal services/	37
37	malpractice/	28036
38	liability, legal/	15815
39	ownership/	9226
40	(law* or legislat* or legal* or medico-legal or medicolegal or statut* or bylaw or by-law* or court* or litigat* or juris* or constitution* or contract or contracts or contractual*).ti,ab,kf.	359878
41	(confidentiality or (confidential adj3 information)).ti,ab,kf.	12509
42	(privacy adj2 (data or genetic* or patient* or health)).ti,ab,kf.	3523
43	(consent* adj2 (third-part* or informed or minor* or parent* or spous* or communit*)).ti,ab,kf.	43919
44	(intellectual propert* or patent* or trade secret* or (propert* adj2 right*)).ti,ab,kf.	58492
45	copyright*.ti,kf.	368
46	(liability or liabilities or tort or torts or tortious or malpractice or negligen*).ti,ab,kf.	35518
47	(treaty or treaties).ti,ab,kf.	2011
48	human rights/	14839
49	civil rights/	10086
50	patient rights/	7150
51	right to health/	147
52	(right* adj2 (civil or human or patient* or health or healthcare or minorit* or equal* or collective)).ti,ab,kf.	33791
53	government regulation/	21607
54	(oversight adj2 government*).ti,ab,kf.	134
55	(regulat* adj3 (government* or federal* or provincial* or state or oversight or requirement* or framework* or guideline* or authorit* or agenc* or body or bodies or data or device* or health or healthcare or medical or approval* or compliance or hurdle* or obstacle* or barrier* or issue*)).ti,ab,kf.	71321
56	(regulations or regulatory environment*).ti,ab,kf.	49909
57	(guidances or guidance document*).ti,ab,kf.	2188
58	or/1-16	215779
59	or/17-57	711869
60	58 and 59	5126
61	limit 60 to yr="2012 -Current"	3918

Searches will be conducted between 2012 and the start of the review. This decision was made through consultation with all team members and was made to account for more recent developments in the field of AI and the deployment of AI into healthcare settings. For example, the development of deep learning AI is viewed as a “paradigm shift” in the field [22]. While deep learning AI was developed prior to 2012, the use of deep learning in the ImageNet Large Scale Visual Recognition Challenge 2012-winning AlexNet [23] led to the technology’s increased public recognition. Experts accordingly view 2012 as the year deep learning was “widely accepted as a viable form of” AI [2]. Health-related AI has become more common since that date [2].

Several members of the research team were part of a previous Royal College of Physicians and Surgeons of Canada Task Force Report on Artificial Intelligence and Emerging Digital Technologies (TH, DS, CF) that contained a bibliography on the grey literature [10]. The team will use that bibliography as a starting point for identifying relevant grey literature, supplementing it by crowdsourcing information about subsequent grey literature among experts working on the research project and further hand-searching, including review of references for mentions of other reports by governments (e.g., [24]), non-governmental organizations (e.g., [4, 12]), and professional organizations (e.g., [10]). Initial examinations of such reports suggest that they are written from an interdisciplinary perspective and thus inappropriate for our task of mapping concerns across disciplines. However, we will summarize their statements on legal concerns and compare them with those in published records.

### Stage three: Study selection

#### Eligibility criteria

Our search strategy intends to identify published literature. It will be designed to identify indexed records, including articles and book chapters. It will also include American law journals, which are academic journals that undergo a standard student review process. Publications will not be limited to works in law journals as the reviewers are interested in what people working in other fields view as key legal concerns (or if they are even discussing legal concerns). Articles in medical, social scientific, computer science or engineering journals that raise legal concerns or solutions thereto are included in this review. We will not include abstracts and conference proceedings.

This review is centrally focused on identifying records that describe, articulate, analyze, or prioritize legal concerns associated with health-related AI. It is specifically focused on identified concerns in health-related AI that may require a legal response. This could include records identifying concerns that require a clear legal solution (e.g., a legal gap), evaluation of existing laws to meet the concerns, and examining the nature, advantages, and disadvantages of various law reform proposals. We will include records addressing various legal concerns (e.g., an omnibus of concerns requiring legal responses from bias to liability) as well as those addressing a single concern (e.g., privacy). We will exclude records that simply describe existing law(s) that apply to the use of health-related AI. We recognize that “law” and “ethics” are sometimes conflated and overlap in some respects. Yet, ethical concerns are and should be distinguished from legal concerns: not all laws reflect our best moral standards and not all ethical concerns implicate the law. Further, ethical debates about health-related AI have been the subject of a scoping review [5]. To this end, records that identify an ethical concern will only be included when there is clearly an articulated nexus to a legal issue (for example, where there is an ethical concern related to privacy and then whether privacy laws are sufficient to protect patients and providers when using ML devices).

Records will be eligible for inclusion if they are English- or French-language publications that describe a legal concern pertaining to the use of AI that is health-related. Working definitions for the key terms (“legal concerns,” “AI,” and “health-related”) are summarized succinctly in Table 2. For the initial searches, we will not impose a language restriction. However, we will include only records published in English or French in the scoping review itself.

**Table 2:** Key terms defined for eligibility assessment

Legal Concerns	Law is “the formal rules of a country passed by a government or its delegated representatives to regulate conduct” [25, 26]. This encompasses formal laws (e.g., constitutions, statutes, the common law) and regulations (e.g., rules passed pursuant to statutory authority). ‘Law’ here does not include ‘soft’ law (e.g., professional college rules). A ‘legal concern’ is one that is identified as requiring a formal governmental response.
Artificial Intelligence (AI)	Per the World Health Organization ([13]: xi, 4), ‘artificial intelligence’ (AI) is “the ability of algorithms encoded in technology to learn from data so that they can perform automated tasks without every step in the process having to be programmed explicitly by a human” and as the “performance by computer programs of tasks that are commonly associated with intelligent beings.” AI, then, refers to machines that can perform acts that typically require human cognition without direct human assistance. This covers a range of tools including those that read medical images to possible future surgical robots. AI does not include electronic tools that merely aid in data collection that do not have an associated AI component (e.g., wearable sensors, computer-assisted decision supports).
Health-Related	AI in this review is ‘health-related’ if it pertains to “healthcare” or “public health.” Healthcare is understood as “efforts made to maintain or restore physical, mental, or emotional well-being especially by trained and licensed professionals” [27]. This also includes activities by trainees or AI where the trainee or AI functions in the same capacity as the licensed professional. “Public health” is understood as “the art and science dealing with the protection and improvement of community health by organized community effort and including preventive medicine” [28]. This definition focuses on activities typically performed by health professionals (and those serving their functions) and the organization of the healthcare settings in which they work. It includes activities in and the regulation of hospital, physician, long-term care home, and other healthcare provider settings as well as at-home goods and services for curative, diagnostic, and preventative purposes. For greater certainty, it includes activities performed by healthcare professionals and basic features of healthcare systems and their regulation (e.g., rationing decisions, insurance).

#### Eligibility assessment

An eligibility assessment tool (for level 1 title and abstract screening and level 2 full-text screening) will be developed and pilot tested in collaboration with the entire review team. Using a proprietary review software program to manage records, duplicate assessment of each record to determine inclusion will be conducted independently. Agreement will be assessed and reported using a Kappa statistic (e.g., [29]). Conflicted decisions will be resolved by one of the subject matter expert authors (MD, CF, DS). Eligibility assessment and flow of the review will be reported in accordance with the PRISMA flow diagram.

### Stage four: Charting the data

A standardized form to extract and categorize data will be developed through a series of consultations with the team and pilot-tested using known includes. The form will cover two primary areas of information: (1) record-level demographic characteristics and (2) content specific to legal concerns, priority areas, solutions, etc., that can be inferred from the records.

Basic record-level information will include year of publication, disciplinary area of focus (e.g., law, medicine), geographic information (e.g., country, region), publication type, study design, language of publication, and area of health-related focus. For example, we will identify the medical specialty in which AI and the legal concern are being confronted. To categorize, we will design a list of specialties (using as a base the list employed by the Royal College of Physicians and Surgeons of Canada but modifying it to, e.g., add family medicine specializations). When this list is insufficient to capture the area of focus (e.g., general medicine, medical administration, electronic health record), we will extract the information verbatim as described in the original record. Given that this is a scoping review and is meant to be iterative and that information gathered can inform future stages, any additional decisions on data to be extracted that occur beyond the protocol stage will be declared transparently in the distal publication.

When extracting data pertaining to any legal concern(s), we will also extract any specific text that expressly indicates prioritization of the concerns and proposed solutions (e.g., new or modified regulations), including new interpretations or expansions of private law responsibilities (e.g., in tort law or contract law), ethical reform (e.g., on the part of AI innovators), and educational/training reforms. When looking at the prioritization of legal issues, we will rely on explicit self-identified priority claims (describing issues as “high priority,” “most important,” “most pressing,” etc.).

### Stage five: Collating, summarizing, and reporting results

Once data has been extracted and accuracy verified through duplicate assessment, we will summarize all record-level characteristics and use the summaries to map publication patterns (who is publishing on the intersection of health related-AI and law) and thematically code legal concerns into categories (e.g., typology). We will categorize the legal concerns using our framework, which seeks to summarize the legal concerns, priorities, and proposed solutions to the concerns.

Once categorized, we will examine the extent to which we see different legal issues of concern to different disciplines (law, medicine, nursing, pharmacy, other health professions, public health, computer science, and engineering). For example, we may see that privacy is raised as a concern by those in the fields of computer science or engineering whereas liability may be more frequently raised as a concern by legal researchers. We will also examine whether we see differences in what legal concerns are identified by specialty, country/region, and whether or not any author on the record is identifiable as an expert from a relevant discipline other than that of the primary author. To achieve the latter, we will categorize authors using record-level information pertaining to their institutional affiliation (e.g., School of Engineering) or listed credentials. It is, of course, possible that individuals trained in one discipline work in another discipline (e.g., a lawyer could work in bioethics within a faculty of medicine). However, departmental affiliation is a useful marker of disciplinary perspective and what they represented as a co-author on the work. Any normative recommendations in the article stemming from data collected in this scoping review will be based on our multidisciplinary team’s views on any inferences that can be drawn from the descriptive findings. In accordance with the PRISMA-Scr [30], we will not formally assess methodological quality of records.

### Stage six: Stakeholder consultation

Understanding the complexity of searching and breadth of the review scope, we plan to engage key consumers and stakeholders to suggest additional records and sources when an initial full-text include list is generated. Given that this review is one component of a larger Canadian Institutes of Health Research-funded project, we plan to solicit input from the broader multidisciplinary team, which includes patient partners and international experts on law, AI, and medicine [31]. If gaps in expertise or perspectives are identified, the team will employ a snowball sampling technique whereby an existing contact will be asked to provide the name(s) of other relevant experts and the process can be repeated as needed. The process of selection and consultation will be detailed sufficiently to ensure transparency.

### Limitations

This scoping review is mapping records from across several disciplines and aims to identify legal concerns that are discussed in health-related AI. Given the complexity of searching, and despite all attempts to be comprehensive, it is conceivable that our search strategy may miss relevant records. This may be due to the records using idiosyncratic language or irregular key word entries or other indexing-related barriers. It is also possible that relevant legal concerns could be in records that predate 2012. This said, our team anticipates that important debates will be captured. Finally, it is possible that few works will make explicit claims about the priority of legal issues; however, we will be able to impute the importance of particular legal questions by looking at the number of times that specific legal issues are raised.

## Discussion

Health-related AI offers numerous potential benefits but also several potential pitfalls. Apt regulation is necessary to maximize its potential benefits while minimizing its risks. Regulatory reforms are more likely to maintain the requisite balance when informed by an understanding of how experts prioritize the concerns and potential solutions they have identified. This scoping review accordingly aims to systematically, comprehensively, and cohesively map the extant literature on legal concerns in health-related AI. A comprehensive map of how different disciplines frame, prioritize, and analyze legal concerns in health-related AI and how they propose to address those concerns will support cross-disciplinary understanding of perceived legal barriers or gaps. It will also aid in policymaking through a holistic view of legal concerns in health-related AI and multiple perspectives on solutions. Our review will thus provide the bedrock from which to better develop domestic and international responses to legal challenges posed by health-related AI.

## Data Availability

Not applicable
